# The role of social support and social networks in smoking behavior among middle and older aged people in rural areas of South Korea: A cross-sectional study

**DOI:** 10.1186/1471-2458-10-78

**Published:** 2010-02-18

**Authors:** E Hwa Yun, Yoon Hwa Kang, Min Kyung Lim, Jin-Kyoung Oh, Jung Min Son

**Affiliations:** 1 National Cancer Control Institute, Korea National Cancer Center, 323 Ilsan-ro, Ilsandong-gu, Goyang-si, Gyeonggi-do, 410-769, South Korea; 2 Department of Public Health Service, District Health Care Team, Korean Health Industry Development Institute, Daesung Building, 311-27, Noryangjin-dong, Dongjak-Gu, 156-050, Seoul, South Korea; 3 Department of Social Welfare, Dongguk University, 26, Pil-dong 3-ga, Jung-gu, 100-715, Seoul, South Korea

## Abstract

**Background:**

Although the number of studies on anti-smoking interventions has increased, studies focused on identifying social contextual factors in rural areas are scarce. The purpose of this study was to explore the role of social support and social networks in smoking behavior among middle and older aged people living in rural areas of South Korea.

**Methods:**

The study employed a cross-sectional design. Participants included 1,057 adults, with a mean age of 60.7 years, residing in rural areas. Information on participants' tobacco use, stress, social support, and social networks was collected using structured questionnaires. The chi-square test, the t-test, ANOVA, and logistic regression were used for data analysis.

**Results:**

The overall smoking prevalence in the study was 17.4% (men, 38.8%; women, 5.1%). Overall, stress was high among women, and social support was high among men. Smokers had high levels of social support (t = -2.90, p = .0038) and social networks (t = -2.22, p = .0271), as compared to non- and former smokers. Those in the high social support group were likely to be smokers (AOR = 2.21, 95% CI 1.15-4.26). Women with moderate social ties were less likely to smoke (AOR = 0.18, 95% CI 0.05-0.61).

**Conclusion:**

There was a protective role of a moderate social network level among women, and a high level of social support was associated with smoking behaviors in rural areas. Findings suggest the need for a comprehensive understanding of the functions and characteristics of social contextual factors including social support and social networks in order to conduct more effective anti-smoking interventions in rural areas.

## Background

The prevalence of smoking among Koreans aged over 19 years is 25% [1]. It has been shown tendency to be decreased continuously the smoking prevalence, especially among Korean men. However, the prevalence of smoking remains high (45.1%) [1]. Additionally, the economic burden of smoking in Korea in 2007 has been estimated at approximately 2.1 billion dollars (with 1 dollar equal to 1,000 won), corresponding to 0.29-0.35% of the Korean GDP [2]. In 2005, Korea ratified the framework convention on tobacco control (FCTC). Various tobacco policies and projects have accompanied this framework, including increased taxes, setting the clean air indoor place, services to stop smoking, anti-smoking campaigns, and so on.

Previous studies have reported that the impacts of tobacco price and taxation, smoking cessation mass media campaigns, and sales restrictions differ by gender, education level, occupational status, and smoking status [3-5]. It has been proposed that these various responses to tobacco policy are due to a lack of consideration of the social context [3]. Therefore, previous studies have suggested that in order to strengthen and improve the effect of tobacco policies, it is necessary to develop a comprehensive understanding of smoking behavior by considering the social context [4,5].

It is well known that smoking behaviors are related to psychosocial factors, including stress, social support, attitude and belief toward smoking behavior, social norms, and social networks [6-11]. Among these factors, stress plays an important role in the urge to smoke, and social support and social networks have been established as moderators of stress [12-14]. Additionally, previous studies have reported that social support and social networks are directly associated with health outcomes and health behavior [15,16]. In fact, it has been reported that smoking cessation intervention and alcoholics anonymous using social support groups were effective [17,18]. In addition, social support has been associated with successful attempts at smoking cessation and prevention of relapse [17,19,20].

Social support and social networks can be defined as sub-concepts of social relations [21]. In other words, social support is a function of social relations provided by members within a social network, and social networks generally relate to the number or contact frequency of family members, relatives, friends, and colleagues [15,21]. Beliefs and attitudes toward smoking, as well as information and social norms about tobacco use influence smoking behavior and may be formulated through interactions with members of the social network. Furthermore, because the social network functions as a social support resource, support concerning smoking behavior can influence a member's smoking behavior [22]. In fact, a previous study describes the social network as another form of structural social support [18]. It has been reported that social networks and social support interact with each other and are positively associated with health behaviors [23]. However, a previous study in Sweden reported that social participation does not always enhance health-related behaviors. Therefore, identification of the pathways by which social contextual factors such as social support and social networks are associated with smoking behavior may aid in the development of effective tobacco policies and smoking cessation intervention programs.

In Korea, studies have focused on the change in smoking prevalence, the evaluation of smoking prevention and smoking cessation, and tobacco policies [24]. However, studies that focus on identifying the social contextual factors are scarce. Additionally, the majority of studies have been conducted in urban areas, and the majority of study participants have been adolescents or adults [24,25]. Adults who live in rural areas have likely been overlooked in smoking-related studies. Therefore, the current study was conducted with the aim to explore the role of social support and social networks on smoking behavior among middle and older aged people living in rural areas of South Korea.

## Methods

### Study design and procedure

This is a cross-sectional study which was conducted at two of southern rural areas in Korea. From 2 to 11 July 2007, data were obtained from the baseline survey for community-based cohort study in rural areas carried out by the Korean National Cancer Center. Public health center in study area, which administered by government, and heads of village in each area were cooperated with the study team to make sure the importance of the study and encourage participation. 1,116 adults aged over 30 years voluntarily participated in the study among 10,905 eligible population (5,017 men and 5,888 women), and 1,057 (387 men and 670 women) subjects who complete the questionnaire on basic demographic characteristics, smoking, stress, social support, and social network were included final analysis. Participants were interviewed face-to-face by research assistants who were trained nursing students from a local nursing school. Information on smoking status, stress, social support, social network, demographic and socioeconomic factors, and other confounding factors was collected. Participants were asked to complete a survey questionnaire that required approximately 40 minutes. Upon completion, the participants were given a gift as a token of appreciation. The institute of research board of the Korea National Cancer Center approved the study, and written informed consent was obtained from each participant.

### Measures

#### Smoking status

Smoking status was measured by an interviewer-administered questionnaire. A current smoker was defined as a subject who reported smoking at least 400 cigarettes during his/her lifetime and who smoked at the time of the survey. Past smokers were defined as persons who reported smoking at least 400 cigarettes during their lifetime, but did not smoke at the time of interview. The remaining participants were defined as non-smokers.

#### Stress

Stress was assessed using the psychosocial well-being index - short form (PWI-SF), which was developed by Chang (2000) [26]. The PWI-SF consists of 18 items that are each scored on a four-point Likert scale. The total PWI-SF score, which ranges between 0 and 54, is calculated by summing the 19 scores. A subject with a higher score experiences more stress. Subjects with the score under 9, 9 to 27, and more than 27 were classified as low, moderate and high stress group, respectively.

In addition, four groups were categorized based on the quartile score. A high score indicates higher stress. Cronbach's alpha for the total PWI-SF scale was 0.88 in this study.

#### Social support

Social support was assessed using the medical outcome study - social support survey (MOS-SSS), which was developed by Sherbourne and Stewart (1991) [27]. The MOS-SSS consists of 19 functional support items that are hypothesized to measure four dimensions of social support: (1) emotional/informational support (the expression of positive affect, empathetic understanding, and the encouragement of expression of feelings/the offering of advice, information, guidance, or feedback), (2) tangible support (the provision of material aid or behavioral assistance), (3) positive social interaction support (the availability of other persons to do fun things with you), and (4) affectionate support (involving expressions of love and affection). A score for each social support scale was computed by averaging across items. Scales were then transformed so that the lowest possible score was 0 and the highest possible score was 100. In addition, four groups were created based on the quartile score. The higher score group indicates the high social support group. Cronbach's alpha for the total MOS-SSS scale was 0.98 in this study.

#### Social network

Social networks were assessed using the social network index (SNI) based on the index developed by Berkman (1979) [28]. The eTheThe The SNI consists of four domains: marriage or partnership, friends and relatives, religious activity, and voluntary associations. Categories were scored as follows: married (married = 1, widowed, divorced, separated, or never married = 0); contact with friends and relatives (≥156 contacts per year = 1, <156 contacts per year = 0); frequency of church or religious service attendance (attended four or more services per year = 1, attended less than four = 0); group (such as church groups, unions, fraternal or athletic groups, or school groups) participation (yes = 1, no = 0). Scores were summed for the four dichotomized variables with a range of 0 to 4. Scores of 0 and 1 indicate the fewest ties and a score of 4 the most ties. Cronbach's alpha for the SNI scale was 0.25 in this study.

#### Other confounding variables

The questionnaire on alcohol intake included alcohol drinking status (non-, former, or current drinker), duration of alcohol drinking, age at start of alcohol drinking, weekly number of drinks, and types of alcoholic beverage or groups of beverage. Lifetime alcohol intake was estimated by multiplying the duration of alcohol intake, weekly number of drinks, and ethanol amount by types of alcoholic beverage. Lifetime alcohol intake was divided into three groups (never, less than 24 g per day [mild or moderate drinker], and more than 24 g per day [heavy drinker]) [29]. Body mass index (BMI) was calculated based on height and weight.

### Data analysis

Data analyses were performed using SAS 9.2. First, the distributions of demographic factors, socioeconomic factors, behavioral factors, and psychosocial factors by smoking status were analyzed. The chi-square test, the t-test, and ANOVA were used to examine associations between the major factors and smoking. Multivariate analysis was performed using ANOVA and a logistic regression model to investigate the impacts of psychosocial factors on smoking, considering possible confounders. All analyses were stratified by sex and smoking status.

## Results

### Demographic characteristics

The demographic characteristics are presented in Additional file 1. The participants (n = 1,057) ranged in age from 30-84 years, with a mean age of 60.7 years. The mean age did not differ between genders. The majority of the participants graduated high school (80.1%), and had a blue-collar job (95.9%) that occupied agriculture. Of the participants, 75.2% had a partner (men, 92.8%; women, 65.4%). Among the participants, 17.4% (men, 38.7%; women, 5.1%) were current smokers, and 40.3% (men, 66.1%; women, 25.4%) were current drinkers.

The distributions of the smoking-related psychosocial and social contextual factors are presented in Additional file  1: table s2. The proportion with high stress was 19.8% (men, 11.1%; women, 24.8%), and stress significantly differed by gender. Women had higher stress as compared to men. In this study, participants had high scores for social support, with a mean score of 78.4 (SD 22.9). The value of social support was significantly higher for men (mean 83.1, SD 20.2) than for women (mean 75.7, SD 24.0). In addition, men reported higher social support in all four sub-domains of social support, as compared to women. The majority of participants maintained moderate ties with family, friends, and relatives, but women appeared more likely to be isolated as compared to men. The number of close families and friends and the frequency of contact with family and friends were similar between men and women, but men participated in a significantly greater number of social groups.

### Stress, social support, and social networks by smoking status and gender

Additional file  1: table s3 shows the distribution of stress, social support, and social networks by smoking status and gender. Differences in stress, social support, and social networks by smoking status and gender were identified. However, the number of female current smokers was small. Therefore, the gender differences in social contextual factors by smoking status could be biased.

The psychosocial stress level was higher in non- and former smokers than in current smokers (t = 2.65, p = .0081), but did not differ by smoking status within the gender groups (F = 20.85, p < .0001). Generally, social support levels were higher among current smokers than non- and former smokers (t = -2.90, p = .0038), and this also did not differ by smoking status within the gender groups (F = 8.96, p < .0001). However, social support levels of female non- and former smokers were significantly low as compared to men of all smoking statuses. The results of tangible social support, positive interaction support, emotional and informational support, and affectionate support as sub-domains of social support evidenced similar patterns to that of general social support.

Social networks were evaluated by the number of close family members and friends, the frequency of contact with family and friends, and the level of social participation. Current smokers were evaluated as having a good social network as compared to non- and former smokers (t = -2.22, p = .0271). Female non- and former smokers had greater social networks than male and female current smokers (F = 14.31, p < 0001). The social network level among men did not differ by smoking status, but that of women significantly differed by smoking status. Female current smokers had the lowest social network levels. Specifically, the number of close family members and friends and the frequency of contact with family and friends did not differ by smoking status or gender. However, social group involvement showed differentiation by gender and by smoking status among women (F = 23.63, p < .0001).

### The effects of stress, social support, and social networks on smoking behavior

The effects of stress, social support, and social networks on smoking status are presented in Additional file  1: table s4 and were evaluated considering confounding factors including demographic characteristics, socioeconomic status, and other health-related factors.

Stress had no effect on smoking status for either gender. Females that were fully supported by family, friends, and neighbors were likely to be current smokers (AOR = 3.77, 95% CI: 1.24-11.48), but other groups such as male current smokers, male non- and former smokers, and female non- and former smokers were not affected by social support. Among the four sub-domains of social support, those in the high positive social interaction group were more likely to be current smokers (AOR = 2.21, 95% CI: 1.15-4.26) as compared to those in the low positive social interaction group. Additionally, men in the high positive social interaction group had a tendency to be current smokers (AOR = 2.26, 95% CI: 1.06-4.82), but this was not the case for women. The social network effect on smoking status was significant among women in the moderate ties group, but not among men.

## Discussion

The results of this study showed significant differences in social contextual factors between non- and former vs. current smokers and different patterns by gender. Inconsistent with other studies on the older population and general population [9,12,30,31], the stress level of current smokers was not higher than that of non- and past smokers, and a moderate social network level significantly influenced smoking behaviors among women only. In addition, a high level of social support was positively related to smoking.

The smoking prevalence in this study reported 17.4%. This prevalence was quite lower compared to that among Koreans aged over 19 years who was about 25% [1]. However, mean age of this study participant was 60 years old and lived in rural area. And other study on the relations of social support to the health behaviors and health status in elderly was surveyed the smoking behavior for 8,688 elderly people, and that smoking rate was 18.6% [32]. Therefore, once considering the demographic characteristics of study participants, this smoking prevalence seemed not quite low.

There were few studies on why is the difference of smoking prevalence between urban and rural population in Korea. However, recently reported study showed that the inequality of smoking prevalence by age and education level have been increased, but the inequality of smoking prevalence by residence area using location quotient was not a difference after controlling the socio-economic characteristics at individual level [33]. Results of the inequality of smoking prevalence considered socio-economic characteristic, but not smoking related other psychosocial and health status. And other daily living pattern, which is different between urban and rural area, could be considered such as leisure time activity, working content & time, social relationship, and so on. Therefore, regarding lack of studies in these issues, further studies are recommended for evident explanation.

Previous studies have been reported that smoking behavior is affected by stress as the response to interact with a social-environmental context even though perceived subjective stress [34,35,12], and social support regulates the effect of stress on smoking behaviors [36]. However, the result of this study was inconsistent with previous studies. The finding showed that the level of stress in non- and former smoker was significantly higher than that in current smoker, but that the relationship of smoking behavior and stress was no longer significant once demographic, psychosocial, and other factors had been adjusted statistically, including gender, age, education level, family income, BMI, alcohol intake, social support and social network. The high level of social support had likely to be more smoke, contrary to expectation. These findings suggest the possibility that smoking as a means for reducing stress could be used, which might be encouraged by families or friends who smoke [36,37,32].

Social support as a function of social relation has been introduced and recognized as a facilitator for improving health behavior [15]. The size of the social network as a resource of social support positively affects health behavior, especially among older people [15,38]. However, other studies have focused on the closeness within social network members and have pointed out that high homogeneity of a social network could reinforce smoking [22]. Furthermore, social networks change over time, and older people have a tendency to construct a social network with close social partners [39]. The result of this study that a high intimacy level of social network did not serve a protective role for smoking but, rather, that a moderate social network level played a protective role for smoking is in partial agreement with studies on Korean older populations [32,40].

Social support has been defined as a facilitator for reaching goals with others and then induced the change of situation and as a qualitative aspect of social relations [18,41]. Generally, social support has been recognized as a moderator of stress and has been used in interventions such as smoking cessation and alcohol abuse [18,42]. In fact, group intervention for smoking cessation using social support group has been shown to be more effective [43]. However, the current study showed that those in the high-level social support group were likely to be current smokers. This result is in disagreement with previous studies, but consistent with some studies in South Korea [32,40]. Smokers have a tendency to continue their smoking behavior as a result of rationalizations about the benefit of smoking filtered cigarettes [44,45]. Social norms about smoking and attitudes toward smoking as a product of interaction with members within the social network may reinforce smoking behavior [16]. In addition, smokers have a high degree of knowledge about the health risks of smoking, but a low will to quit smoking [46]. Positive social norm about smoking may be influenced by self-appraisal of benefit-harm on smoking, smoking acceptability within social network, delivering the misinformation by significant others, and so on [47,27]. Also, relatively low price of tobacco in Korea could be contributed to smoke in that make to improve the accessibility of social resource availability as part of social support [48]. The negative effect of social support on smoking behaviors in this study can be explained by the older age of the participants and the results of interactions between social support and social networks in rural areas [49]. These results suggest that in order to improve the effect of smoking-related interventions or policies, especially in rural areas, it is necessary to identify the features of social support and social networks.

Various smoking cessation interventions have been performed. Among them, group behavioral intervention and group counseling have been taken up as effective programs except population-based mass media campaigns [50,51]. The advantage of group intervention or counseling is that it strengthens the will to quit and induces the motivation to quit by changing misconceptions, beliefs, and attitudes about smoking through participant interaction [52]. Therefore, simple stay in grouping intervention or counseling is hard to increase the quitting rates. To improve the quitting rates of smokers, especially older smokers, interaction among participants should be activated. Social contextual factors such as social support and social networks should be used as a pathway to activate interaction among smokers. However, social contextual factors may differ by age, gender, education level, and living area. A previous study showed that women with a low level of social network had a tendency to be smokers [49]. In fact, this study found a difference in psychosocial factors and social contextual factors by gender and smoking status, with men in the high positive interaction support group being more likely to smoke, but not women. These results provide evidence that a specific approach for quitting by gender using the pathway of social support and social networks is needed.

A study on the functions of the social networks of rural elders in Korea pointed out that although elders recognize their sons and daughters as important members of their social network, friends and neighbors function as the most influential aspects of the social network in daily living [53]. In fact, the duration of acquaintance with neighbors was more than ten years for half of the elders in rural areas [53]. This indicates that the characteristics of rural elders' social networks could transition from heterogeneity and peripheral partners to more homogeneity and closer partners over time [54]. Smoking pattern by the level of social network can be explained by aspect of social activity, partially. This suggest the possibility that women smoker with high social network could have or make many opportunity to be smokers as intimated networkers, whereas women smoker with low social network may rather go out for smoking with intimated networkers than regulate their stress with smoking alone. In Korea, these patterns have been formed by lesser reluctant environment for smoking of elderly women than that of younger women. Therefore, a high level of social network ties may not serve a protective role in smoking among women. The result of this study is consistent with that of previous studies that have reported that poor social networks among women are associated with smoking behavior [47,49]. In addition, this finding provides evidence that it is necessary to sustain the optimal social network level not to smoke in women [49].

Social networks can be defined as a resource of social support, a type of function of social relations, and objective informal social support [39,55,56]. A study on HIV risk behaviors described the characteristics of social networks and showed that social networks that consisted of persons with risky health behavior had a tendency to decrease in size and that localized social networks play a central role in unknowingly spreading health risk behaviors [57]. This feature of social networks can be applied to delivering a smoking intervention program by identifying high-risk groups [58]. As tobacco policies such as clean air ordinances and home restriction have increased, smokers are likely to be isolated from public places [59]. Therefore, smoking social networks may be localized and stronger. In this study, the findings of poor social networks and high social support among female smokers suggest that this feature of social networks in rural areas in Korea may be similar. Therefore, in rural areas, identification of smoking social networks including smoking-related risk groups should precede anti-smoking interventions.

A limitation of this study is that social norms, attitudes, and beliefs about smoking behavior were not assessed. This study could not identify the social norms and attitudes toward smoking behavior as a product of interaction among social network members. A comprehensive understanding of the relationship of social support and social networks is difficult to achieve due to a lack of information on them. Therefore, future studies should be conducted to identify the relationship between social contextual factors and high-risk behavior among elderly rural persons.

Other limitation is the small number of women smoker to make a comparison with the influence of psychosocial factors. Although the smoking prevalence of women in this study was not low, the number of women smoker included in each group, which was divided by the level of stress, social support, and social network, was not enough to be compared with non- and former-women smoker. Therefore, the effect of social network in women may be biased result. To get a more information about the interaction among smoking behavior, stress, social support, and social network in detail should be considered the low smoking rate in women in Korea.

For the limitations on representativeness of the study subjects and generalization of our findings exist. We recruited voluntary participants in study areas without any randomization and/or stratification considering age and gender distribution of eligible population. Therefore, some selection bias and difficulties to generalize our finding could be. Comparing the age and gender distribution between study subjects and eligible population, there is not much difference, even though proportion of female and proportion of aged under 40 years in study subjects is relatively higher and lower than those in eligible population, respectively. However, these issues are usually happened in many other community based survey, and the proportion of old aged subjects in study population was similar with that in eligible population. In addition, many of middle aged residents who were registered in rural areas might not reside in their registered habitat, because they usually live in other areas for job seeking or convenience of daily living. These situations could affect to lessen the actual number of middle aged subject we can contact in the study areas.

## Conclusion

There is preliminary evidence that social support and social networks influence smoking behavior among rural persons of South Korea. This study showed that identification of the pathway of social support and social networks may aid in the effective delivery of anti-smoking interventions, especially among rural elders. Smoking related to social influence should be explicitly investigated in longitudinal research and applied tobacco control policy.

## Competing interests

The authors declare that they have no competing interests.

## Authors' contributions

YHK initiated the study, assisted with data analysis, and wrote the article. EHY contributed to the study design and analytic plan, performed data analysis, and revised the final draft of the manuscript. MKL supervised all aspects of the study implementation and contributed to the interpretation and writing of the article. JKO assisted in data collection. JMS assisted with data analyses and provided input on drafts of the manuscript. All authors reviewed drafts of the manuscript and approved the version for publication.

## Pre-publication history

The pre-publication history for this paper can be accessed here:

http://www.biomedcentral.com/1471-2458/10/78/prepub

## Supplementary Material

Additional file 1 Supplementary tables Click here for file

## References

[B1] The Ministry for Health, Welfare, and Family Affair & the Center for Disease Control and Prevention The Korea National Health and Nutritional Examination Survey. 2008 2008 Seoul: The Ministry for Health, Welfare, and Family Affair

[B2] Park SE Song HR Kim CH Ko SK Economic burden of smoking in Korea, 2007 Korean J Health Promot Dis Prev 2008 8 4 219 227 [Korean]

[B3] Greaves L Hemsing N Women and tobacco control policies: Social-structural and psychosocial contributions to vulnerability to tobacco use and exposure Drug Alcohol Depend 2009 104 Suppl 1 S121 30 10.1016/j.drugalcdep.2009.05.001 19520523

[B4] Niederdeppe J Fiore MC Baker TB Smith SS Smoking-cessation media campaigns and their effectiveness among socioeconomically advantaged and disadvantaged populations Am J Public Health 2008 98 916 924 10.2105/AJPH.2007.117499 18381998 PMC2374829

[B5] Gagne L The 2005 British Columbia Smoking Cessation Mass Media Campaign and short-term changes in smoking J Public Health Manag Pract 2007 13 296 306 17435497 10.1097/01.PHH.0000267688.54024.09

[B6] De Vogli R Santinello M Unemployment and smoking: does psychosocial stress matter? Tob Control 2005 14 389 95 10.1136/tc.2004.010611 16319362 PMC1748130

[B7] McCormick MC Brooks-Gunn J Shorter T Holmes JH Wallace CY Heagarty MC Factors associated with smoking in low-income pregnant women: relationship to birth weight, stressful life events, social support, health behaviors and mental distress J Clin Epidemiol 1990 43 441 8 10.1016/0895-4356(90)90132-9 2324784

[B8] Lindstrom M Social capital and the miniaturization of community among daily and intermittent smokers: a population-based study Prev Med 2003 36 177 84 10.1016/S0091-7435(02)00049-X 12590993

[B9] Daniel M Cargo MD Lifshay J Green LW Cigarette smoking, mental health and social support: data from a northwestern First Nation Can J Public Health 2004 95 45 9 14768741 10.1007/BF03403633 PMC6975667

[B10] Steptoe A Wardle J Pollard TM Canaan L Davies GJ Stress, social support and health-related behavior: a study of smoking, alcohol consumption and physical exercise J Psychosom Res 1996 41 171 80 10.1016/0022-3999(96)00095-5 8887830

[B11] Allen J Markovitz J Jacobs DR Jr Knox SS Social support and health behavior in hostile black and white men and women in CARDIA. Coronary Artery Risk Development in Young Adults Psychosom Med 2001 63 609 18 11485115 10.1097/00006842-200107000-00014

[B12] Kang SJ Nam CH Lee CH Kang SU Kim MH Oh SY Lee SH Smoking status of residents in an urban area and affecting variables Kor J Oriental Prev Med Soc 2008 12 3 185 197 [Korean]

[B13] Brennan PL Moos RH Life stressors, social resources, and late-life problem drinking Psychol Aging 1990 5 491 501 10.1037/0882-7974.5.4.491 2278671

[B14] Folkman S Chesney MA Pollack L Phillips C Stress, coping, and high-risk sexual behavior Health Psychol 1992 11 218 222 10.1037/0278-6133.11.4.218 1396489

[B15] Golden J Conroy RM Lawlor BA Social support network structure in older people: underlying dimensions and association with psychological and physical health Psychol Health Med 2009 14 280 290 10.1080/13548500902730135 19444706

[B16] Homish GG Leonard KE The social network and alcohol use J Stud Alcohol Drugs 2008 69 906 914 18925349 10.15288/jsad.2008.69.906 PMC2583378

[B17] Carlson LE Goodey E Bennett MH Tanezer P Koopmans J The addition of social support to a community-based large-group behavioral smoking cessation intervention: Improved cessation rates and gender difference Addict Behav 2002 27 547 559 10.1016/S0306-4603(01)00192-7 12188591

[B18] Groh ER Jason LA Keys CB Social network variables in alcoholics anonymous: A literature review Clin Psyhol Rev 2008 28 3 430 450 10.1016/j.cpr.2007.07.014 PMC2289871 17719158

[B19] Hanson BS Isacsson SO Janzon L Lindell SE Social support and quitting smoking for good. Is there an association? Results from the population study, "Men born in " Malmo, Sweden Addict behav 1914 15 221 233 10.1016/0306-4603(90)90065-6 2378282

[B20] Fisher EB Jr Two approaches to social support in smoking cessation: commodity model and nondirective support Addict behav 1997 22 819 833 10.1016/S0306-4603(97)00064-6 9426800

[B21] Due P Holstein B Lund R Modvig J Avlund K Social relations: network, support and relational strain Soc Sci Med 1999 48 661 673 10.1016/S0277-9536(98)00381-5 10080366

[B22] Vaanamen A Kouvonen A Kivimaki M Pentti J Vahtera J Social support, network heterogeneity, and smoking behavior in women: the 10-town study Am J Health Promot 2008 22 246 255 18421889 10.4278/0701094R1.1

[B23] Lindstrom M Hanson BS Ostergren PO Berglund G Socioeconomic differences in smoking cessation: the role of social participation Scand J Public Health 2000 28 200 208 11045752

[B24] Yang SJ An analysis of trends in smoking-related research J Korea Acad Pub Health Nur 2008 22 2 255 265 [Korean]

[B25] Hyun HJ Ahn HY An analysis of the research on effect of smoking cessation intervention J Korea Acad Comm Health Nur 2008 19 3 469 479 [Korean]

[B26] Chang SJ Standardization of collection and measurement for heath data Kyechukmunhwasa, Seoul 2000 121 59 [Korean]

[B27] Sherbourne CD Stewart AL The MOS social support survey Soc Sci Med 1991 32 705 714 10.1016/0277-9536(91)90150-B 2035047

[B28] Berkman LF Syme SL Social networks, host resistance, and mortality: a nine-year follow-up study of Alameda County residents Am J Epidemiol 1979 109 186 204 425958 10.1093/oxfordjournals.aje.a112674

[B29] U.S. Department of Agriculture (USDA) Nutrition and your health: Dietary guidelines for Americans 2005 Washington: USDA

[B30] Honda K Psychosocial correlated of smoking cessation among elderly ever-smokers in the United States Addict Behav 2005 30 375 381 10.1016/j.addbeh.2004.05.009 15621410

[B31] Brwon C Madden PA Palenchar DR Cooper-Patrick L The association between depressive symptoms and cigarette smoking in an urban primary care sample Int J Psychiatry Med 2000 30 1 15 26 10.2190/NY79-CJ0H-VBAY-5M1U 10900558

[B32] Kim TM Lee SG Jeon SY The relations of social support to the health behaviors and health status in the elderly J Korean Soc Health Edu Promot 2006 23 3 99 119

[B33] Kim HR Kang YH Yun KJ The political recommendation and the difference of health status by socio-economic position 2004 Seoul: Korea institute of health and welfare [Korean]

[B34] Tsai YW Wen YW Tsai CR Tsai TI Peer pressure, psychological distress and the urge to smoke Int J Environ Res Public Health 2009 6 6 1799 1811 10.3390/ijerph6061799 19578461 PMC2705218

[B35] Kassel JD Stroud LR Paronis CA Smoking, stress, and negative affect: Correlation, causation, and context across stages of smoking Psychol Bull 2003 129 270 304 10.1037/0033-2909.129.2.270 12696841

[B36] Conway TL Vickers RR Ward HW Rahe RH Occupational stress and variation in cigarette, coffee and alcohol consumption J Health Soc Behav 1981 22 155 165 10.2307/2136291 7240714

[B37] Brandon TH Baker TB The smoking consequences questionnaire: The subjective expected utility of smoking in college students Psychol Assess 1991 3 484 491 10.1037/1040-3590.3.3.484

[B38] Cornwell EY Waite LJ Social disconnectedness, perceived isolation, and health among older adults J Health Soc Behav 2009 50 31 48 10.1177/002214650905000103 19413133 PMC2756979

[B39] Yeung DY Fung HH Lang FR Self-construal moderates age differences in social network characteristics Psychol Aging 2008 23 222 226 10.1037/0882-7974.23.1.222 18361670

[B40] Lee MS Kim DK Kim EY Na BJ Sung TH A study on the relationship between social support, social network and health behaviors among some rural peoples J Korean Soc Health Edu Promot 2002 29 2 73 98 [Korean]

[B41] Cohen S Underwood LG Gottlieb BH Social Support Measurement and Intervention 2000 New York: Oxford University Press

[B42] Sorensen G Emmons K Hunt MK Barbeau E Goldman R Peterson K Kuntz K Stoddard A Berkman L Model for incorporating social context in health behavior interventions: applications for cancer prevention for working-class, multiethnic populations Prev Med 2003 37 188 197 10.1016/S0091-7435(03)00111-7 12914824

[B43] Seo NS Kim YH Kang HY The effects of a group smoking cessation program among adult smokers in a rural community J Korean Acad Nur 2007 37 7 1139 1148 [Korean] 10.4040/jkan.2007.37.7.1139 18182875

[B44] Cummings KM Hyland A Giovino GA Hastrup JL Bauer JE Bansal MA Are smokers adequately informed about the health risks of smoking and medicinal nicotine? Nicotine Tob Res 2004 6 Suppl 3 S333 340 10.1080/14622200412331320734 15799596

[B45] Peretti-Watel P Constance J "It's all we got left". Why poor smokers are less sensitive to cigarette price increases Int J Environ Res Public Health 2009 6 2 608 21 10.3390/ijerph6020608 19440404 PMC2672354

[B46] Gong YL Koplan JP Feng W Chen CH Zheng P Harris JR Cigarette smoking in China. Prevalence, characteristics, and attitudes in Minhang District JAMA 1995 274 15 1232 1234 10.1001/jama.274.15.1232 7563514

[B47] White KM Wellington L Predicting participation in group parenting education in an Australian sample: the role of attitudes, norms, and control factors J Prim Prev 2009 30 173 189 10.1007/s10935-009-0167-y 19283485

[B48] Revicke DA Mitchell J Social support factor structure in the elderly Res Aging 1986 8 2 232 248 10.1177/0164027586008002003 3738207

[B49] Romano PS Bloom J Syme SL Smoking, social support and hassles in an urban African-American community Am J Public Health 1991 81 11 1415 1422 10.2105/AJPH.81.11.1415 1951797 PMC1405675

[B50] Lemmens V Oenema A Knut IK Brug J Effectiveness of smoking cessation interventions among adults: a systematic review of reviews Eur J Cancer Prev 2008 17 6 535 544 10.1097/CEJ.0b013e3282f75e48 18941375

[B51] Mottillo S Filion KB Bélisle P Joseph L Gervais A o'Loughlin F Paradis G Pihl R Pilote L Rinfret S Tremblay M Eisenberg MJ Behavioral interventions for smoking cessation: a meta-analysis of randomized controlled trials Eur Heart J 2009 30 6 718 730 10.1093/eurheartj/ehn552 19109354

[B52] Scgumann A Stein JA Yllman JB John U Rumpf HJ Meyer C Patterns and predictors of change in a smoking intervention study: latent growth analysis of a multivariate outcome model Health Psychol 2008 27 3 Suppl S233 242 10.1037/0278-6133.27.3(Suppl.).S233 18979976

[B53] Sub SH Lim HK A study of the functions of social network of rural elders living in Chonnam province Rural Soc 2004 14 1 179 203 [Korean]

[B54] Fung HH Stoeber FS Yeung DY Lang FR Cultural specificity of socioemotional selectivity: age differences in social network composition among Germans and Hong Kong Chinese J Gerontol B Psychol Sci Soc Sci 2008 63 P156 164 18559680 10.1093/geronb/63.3.p156

[B55] Phillips DR Siu OL Yeh AG Cheng KH Informal social support and older persons' psychological well-being in Hong Kong J Cross Cult Gerontol 2008 23 1 39 55 10.1007/s10823-007-9056-0 18228121

[B56] Fiori KL Smith J Antonucci TC Social network types among older adults: a multidimensional approach J Gerontol B Psychol Sci Soc Sci 2007 62 6 P322 30 18079416 10.1093/geronb/62.6.p322

[B57] Latkin C Donnell D Celentano DD Aramrattna A Liu TY Vongchak T Wiboonnatakul K Davis-Vogel A Metzger D Relationships between social norms, social network characteristics, and HIV risk behaviors in Thailand and the United States Health Psychol 2009 28 3 323 9 10.1037/a0014707 19450038 PMC2799116

[B58] Blozik E Wagner JT Gillmann G Iliffe S von Renteln-Kruse W Lubben J Beck JC Stuck AE Clough-Gorr KM Social network assessment in community-dwelling older persons: results from a study of three European populations Aging Clin Exp Res 2009 21 2 150 7 19448387 10.1007/BF03325223

[B59] Christakis NA Fowler JH The collective dynamics of smoking in a large social network N Engl J Med 2008 358 21 2249 58 10.1056/NEJMsa0706154 18499567 PMC2822344

