# Supplementary material for: The role of social support and social networks in smoking behavior among middle and older aged people in rural areas of South Korea: A cross-sectional study
Source: BMC Public Health. 2010 Feb 18;10:78. doi: 10.1186/1471-2458-10-78 (PMC2834631; doi:10.1186/1471-2458-10-78)
Supplement: Additional file 1 — Supplementary tables [file 1471-2458-10-78-S1.DOC]

**Table 1. Distribution of demographic characteristics by gender**

|  | Total(n=1057) | | Men(n=387) | | Women(n=670) | | t or  Chi-square | p | |
| --- | --- | --- | --- | --- | --- | --- | --- | --- | --- |
|  | N(%) or  Mean±SD | | N(%) or  Mean±SD | | N(%) or  Mean±SD | |
| Age | 60.7(±11.5) | | 60.54(±10.6) | | 60.8(±29.0) | | -.37 | .7139 | |
| <40 years | 72(6.8) | | 17(4.4) | | 55(8.2) | | .3651 | .5457 | |
| <50 | 134(12.7) | | 54(14.0) | | 80(11.9) | |
| <60 | 242(22.9) | | 103(26.6) | | 139(20.7) | |
| <70 | 369(34.9) | | 140(36.2) | | 229(34.2) | |
| ≥70 | 240(22.7) | | 73(18.9) | | 167(24.93) | |
| Education level(n=1054) |  | |  | |  | | 24.7697 | <.0001 | |
| <High school | 844(80.1) | | 278(72.0) | | 566(84.7) | |
| ≥High school | 210(19.9) | | 108(28.0) | | 102(15.3) | |  |  | |
| Monthly income ($)* | 139.46(±197.8) | | 174.8(±228.1) | | 119.0(±175.0) | | 4.16 | <.0001 | |
| < 300 | 177(16.7) | | 40 (10.3) | | 137 (20.5) | | 44.5076 | <.0001 | |
| 300-890 | 350(33.1) | | 102 (26.4) | | 248 (37.0) | |  |  | |
| 900-1990 | 252(23.8) | | 116 (30.0) | | 136 (20.3) | |  |  | |
| ≥2000 | 278(26.3) | | 129 (33.3) | | 149 (22.2) | |  |  | |
| Job |  | |  | |  | |  |  | |
| White color | 43(4.1) | | 20 (5.2) | | 23 (3.4) | | 1.8924 | .1689 | |
| Blue color | 1014(95.9) | | 367 (94.8) | | 647 (96.6) | |  |  | |
| Marital status(n=1054) |  | |  | |  | |  |  | |
| Coupled | 795(75.4) | | 358(92.8) | | 437(65.4) | | 98.5644 | <.0001 | |
| Single | 259(24.6) | | 28(7.2) | | 231(34.6) | |  |  | |
| Smoking status | |  | |  | |  |  | |  |
| Non-smoker | | 683(64.6) | | 69(17.8) | | 614(91.6) | 474.0757 | | <.0001 |
| Former smoker | | 190(18.0) | | 168(43.4) | | 22(3.3) |
| Current smoker | 184(17.4) | | 150(38.7) | | 34(5.1) | |  |  | |
| Drinking status(n=1056) |  | |  | |  | |  |  | |
| Non-drinker | 555(52.6) | | 77(19.9) | | 478(71.5) | | 228.2541 | .0001 | |
| Former drinker | 75(7.1) | | 54(14.0) | | 21(3.1) | |  |  | |
| Current drinker | 426(40.3) | | 256(66.1) | | 170(25.4) | |  |  | |
| Alcohol intake(g/day)§ |  | |  | |  | |  |  | |
| 0 g/day | 605(57.3) | | 112 (28.9) | | 493 (73.7) | | 289.3138 | <.0001 | |
| <24g/day | 288(27.3) | | 127 (32.8) | | 161 (24.1) | |  |  | |
| ≥24g/day | 163(15.4) | | 148 (38.2) | | 15 (2.2) | |  |  | |
| BMI (kg/m^2^)** | 23.6(±3.20) | | 23.4(±3.04) | | 23.7(±3.30) | | -1.54 | .1234 | |
| < 18 | 20(1.9) | | 11 (2.8) | | 9 (1.3) | | .0016 | .9684 | |
| 18-22 | 458(43.3) | | 160 (41.3) | | 298 (44.5) | |  |  | |
| ≥23 | 579(54.8) | | 216 (55.9) | | 363 (54.2) | |  |  | |

*1,000 won=1$

** The criterion of BMI was based on the definition of the WHO western-pacific region: underweight, <18 kg/m^2^; normal 18-22 kg/m^2^; overweight or obesity, ≥23 kg/m^2^.

Table 2. Distribution of stress and social support characteristics by gender

|  | Total(n=1057) | Men(n=387) | Women(n=670) | t or  Chi-square | p |
| --- | --- | --- | --- | --- | --- |
|  | N(%) or  Mean±SD | N(%) or  Mean±SD | N(%) or  Mean±SD |
| Stress* | 18.7(±9.13) | 15.9(±8.40) | 20.3(±9.15) | -7.77 | <.0001 |
| <9 | 134(12.7) | 71(18.4) | 63(9.4) | 39.2109 | <.0001 |
| <27 | 714(67.6) | 273(70.5) | 441(65.8) |
| ≥27 | 209(19.8) | 43(11.1) | 166(24.8) |
| Social support | 78.4(±22.9) | 83.1(±20.2) | 75.7(±24.0) | 5.10 | <.0001 |
| Tangible | 77.6(±24.7) | 85.1(±20.1) | 73.6(±26.1) | 7.49 | <.0001 |
| Positive social interaction | 79.2(±24.5) | 83.5(±21.4) | 76.7(±25.9) | 4.42 | <.0001 |
| Emotional and informational | 77.8(±24.2) | 81.5(±21.9) | 75.7(±25.2) | 3.73 | <.0001 |
| Affectionate | 78.7(±24.1) | 82.1(±22.2) | 76.7(±24.8) | 3.55 | <.0001 |
| Social network§ | 2.42(±0.85) | 2.62(±0.71) | 2.31(±0.91) | 6.32 | <.0001 |
| 0, 1 (fewest ties) | 153(14.5) | 24(6.2) | 129(19.3) | 35.4717 | <.0001 |
| 2 | 325(30.8) | 107(27.7) | 218(32.5) |  |  |
| 3 | 532(50.3) | 241(62.3) | 291(43.4) |  |  |
| 4 (most ties) | 47(4.5) | 15(3.9) | 32(4.8) |  |  |
| The number of close family members | 2.47(±0.91) | 2.51(±0.79) | 2.44(±0.82) | 1.11 | 0.2683 |
| The number of close friends | 2.31(±0.91) | 2.41(±1.01) | 2.26(±1.07) | 2.24 | 0.0250 |
| The contact frequency of family | 3.61(±1.05) | 3.61(±1.61) | 3.44(±1.70) | 1.59 | 0.1122 |
| The contact frequency of friends | 3.80(±1.67) | 3.71(±1.80) | 3.84(±1.90) | -1.15 | 0.2486 |
| The number of social groups | 1.55(±1.21) | 1.92(±1.19) | 1.33(±1.16) | 7.88 | <.0001 |

*Stress was divided into three groups based on the criteria of the PWI-SF

§Social network was divided into four groups based on the SNI protocol.

Table 3. Stress, social support, and social network by smoking status and gender

|  | Non- and Former smoker (n=873)  (Mean±SD) | | | Current smoker (n=184)  (Mean±SD) | | t | | p |
| --- | --- | --- | --- | --- | --- | --- | --- | --- |
| Stress | 19.04±9,19 | | | 17.08±8.72 | | 2.65 | | .0081 |
| Total social support | 77.45±23.12 | | | 82.82±21.6 | | -2.90 | | .0038 |
| Tangible social support | 75.50±25.08 | | | 81.09±21.64 | | -4.28 | | <.0001 |
| Positive interaction support | 78.29±24.76 | | | 83.33±23.07 | | -2.54 | | .0112 |
| Emotional and information support | 77.16±24.21 | | | 81.01±23.92 | | -1.97 | | .0496 |
| Affectionate support | 77.85±24.17 | | | 82.72±23.12 | | -2.50 | | .0125 |
| Social network | 2.40±0.87 | | | 2.54±0.76 | | -2.22 | | .0271 |
| The number of close family members | 2.46±0.82 | | | 2.50±0.76 | | -0.65 | | .5159 |
| The number of close friends | 2.30±1.05 | | | 2.36±1.07 | | -0.76 | | .4453 |
| The contact frequency of family | 3.50±1.67 | | | 3.55±1.68 | | -0.39 | | .6958 |
| The contact frequency of friends | 3.83±1.85 | | | 3.66±1.92 | | 1.12 | | .2620 |
| The number of social groups | 1.51±1.22 | | | 1.70±1.15 | | -1.86 | | .0638 |
|  | Male (Mean±SD) | | | Female(Mean±SD) | | F | P | Post-hoc |
|  | Non- and Former smoker(n=237) - a | Current smoker (n=150) - b | | Non- and Former smoker(n=636) - c | Current smoker (n=34) - d |
| Stress | 15.94±8.38 | | 15.85±8.45 | 20.19±9.21 | 22.52±7.86 | 20.85 | <.0001 | c, d> a, b |
| Total social support | 82.18±21.04 | | 84.46±18.86 | 75.69±23.62 | 75.61±30.34 | 8.96 | <.0001 | a, b>c |
| Tangible social support | 84.28±20.97 | | 86.50±18.72 | 73.60±25.87 | 74.26±29.83 | 18.94 | <.0001 | a, b>c |
| Positive interaction support | 82.37±22.49 | | 85.34±19.43 | 76.77±25.40 | 74.48±33.88 | 7.06 | 0.0001 | a, b>c |
| Emotional and information support | 80.84±22.27 | | 82.44±21.41 | 75.79±24.77 | 74.72±32.46 | 4.79 | 0.0026 | b>c |
| Affectionate support | 81.22±22.64 | | 83.57±21.60 | 76.59±24.61 | 78.96±29.03 | 4.60 | 0.0033 | b>c |
| Social network | 2.59±0.75 | | 2.67±0.93 | 3.33±0.90 | 1.94±0.98 | 14.31 | <.0001 | c>a, b>d |
| The number of close family members | 2.52±0.80 | | 2.49±0.78 | 2.44±0.83 | 2.59±0.66 | 0.81 | 0.4887 |  |
| The number of close friends | 2.41±0.97 | | 2.39±1.07 | 2.26±1.07 | 2.24±1.07 | 1.69 | 0.1670 |  |
| The contact frequency of family | 3.60±1.59 | | 3.63±1.64 | 3.46±1.69 | 3.18±1.80 | 1.16 | 0.3238 |  |
| The contact frequency of friends | 3.77±1.73 | | 3.61±1.91 | 3.85±1.89 | 3.85±1.99 | 0.67 | 0.5728 |  |
| The number of social groups | 1.92±1.26 | | 1.91±1.08 | 1.36±1.17 | 0.76±0.99 | 23.63 | <.0001 | a, b>c>d |

Table 4. The effects of stress, social support, and social network on smoking status by gender

|  | Total(n=1057) | | | Male (n=387) | | | Female (n=670) | | |
| --- | --- | --- | --- | --- | --- | --- | --- | --- | --- |
|  | N | No. of smokers (%) | OR(95% CI) | N | No. of smokers (%) | OR(95% CI) | N | No. of smokers (%) | OR(95% CI) |
| Stress* |  |  |  |  |  |  |  |  |  |
| <9 | 134 | 31(16.8) | Reference | 71 | 29(19.3) | Reference | 63 | 2(5.9) | Reference |
| <27 | 714 | 125(67.9) | 0.99(0.59~1.65) | 273 | 103(68.7) | 0.93(0.53~1.65) | 441 | 22(64.7) | 2.18(0.44~10.83) |
| ≥27 | 209 | 28(15.2) | 1.13(0.56~2.28) | 43 | 18(12.0) | 1.04(0.45~2.43) | 166 | 10(29.4) | 2.95(0.52~16.83) |
| P for trend |  |  | P=.8161 |  |  | P=.9526 |  |  | P=.9367 |
| Social support* |  |  |  |  |  |  |  |  |  |
| <65 | 261 | 37(20.1) | Reference | 73 | 27 (18.0) | Reference | 188 | 10 (29.4) | Reference |
| <83 | 257 | 33(17.9) | 0.72(0.40~1.30) | 88 | 29 (19.3) | 0.79(0.39~1.60) | 169 | 4 (11.8) | 0.72(0.20~2.60) |
| <100 | 243 | 44(23.9) | 1.34(0.75~2.37) | 80 | 35 (23.3) | 1.20(0.59~2.42) | 163 | 9 (26.5) | 2.27(0.77~6.72) |
| 100 | 296 | 70(38.0) | 1.37(0.79~2.38) | 146 | 59 (39.3) | 1.09(0.57~2.08) | 150 | 11 (32.4) | **3.77(1.24~11.48)** |
| P for trend |  |  | P=.4540 |  |  | P=.9463 |  |  | P=.2919 |
| Social support** |  |  |  |  |  |  |  |  |  |
| Tangible |  |  |  |  |  |  |  |  |  |
| Low | 538 | 73(39.7) | Reference | 147 | 55(36.7) | Reference | 391 | 18(52.9) | Reference |
| High | 519 | 111(60.3) | 0.71(0.39~1.29) | 240 | 95(63.3) | 0.69(0.32~1.49) | 279 | 16(47.1) | 0.63(0.22~1.83) |
| Positive social interaction |  |  |  |  |  |  |  |  |  |
| Low | 572 | 78(42.4) | Reference | 180 | 63(42.0) | Reference | 392 | 15(44.1) | Reference |
| High | 485 | 106(57.6) | **2.21(1.15~4.26)** | 207 | 87(58.0) | **2.26(1.06~4.82)** | 278 | 19(55.9) | 2.82(0.67~11.83) |
| Emotion & informational |  |  |  |  |  |  |  |  |  |
| Low | 519 | 78(42.4) | Reference | 168 | 64(42.7) | Reference | 351 | 14(41.2) | Reference |
| High | 538 | 106(57.6) | 0.78(0.39~1.57) | 219 | 86(57.3) | 0.56(0.24~1.28) | 319 | 20(58.8) | 1.62(0.35~7.51) |
| Affectionate |  |  |  |  |  |  |  |  |  |
| Low | 470 | 67(36.4) | Reference | 148 | 54(36.0) | Reference | 322 | 13(38.2) | Reference |
| High | 587 | 117(63.6) | 1.23(0.64~2.35) | 239 | 96(64.0) | 1.31(0.59~2.92) | 348 | 21(61.8) | 1.25(0.32~4.87) |
| Social network* |  |  |  |  |  |  |  |  |  |
| 0,1 (fewest ties) | 153 | 18(11.8) | Reference | 24 | 6 (25.0) | Reference | 129 | 12 (35.3) | Reference |
| 2 | 325 | 58(17.9) | 0.82(0.42~1.60) | 107 | 44 (41.1) | 1.90(0.63~5.75) | 218 | 14 (41.2) | 0.68(0.28~1.67) |
| 3 | 532 | 98(18.4) | 0.60(0.31~1.19) | 241 | 93 (38.6) | 1.83(0.62~5.45) | 291 | 5 (14.7) | **0.18(0.05~0.61)** |
| 4 (most ties) | 47 | 10(21.3) | 1.39(0.50~3.90) | 15 | 7 (46.7) | 2.97(0.67~13.12) | 32 | 3 (8.8) | 1.55(0.32~7.64) |
| P for trend |  |  | P=.2299 |  |  | P=.7516 |  |  | P=.2619 |

*Adjusted for sex, age(<40 years, <50, <60, <70, ≥70 years), education level(<high school vs. ≥high school), family income(<30, 30-89, 90-199, ≥200), BMI(<18,18-22, ≥23), alcohol intake(0 g/day, <24 g/day, ≥24 g/day), social support(four groups by quartile), social network(four groups by SNI protocol) when appropriate.

**Adjusted for sex, age(<40 years, <50, <60, <70, ≥70 years), education level(<high school vs. ≥high school), family income(<30, 30-89, 90-199, ≥200), BMI(<18,18-22, ≥23), alcohol intake(0 g/day, <24 g/day, ≥24 g/day), stress (low, moderate, and high stress), social network(four groups by SNI protocol) when appropriate.
